# Shared attention in virtual immersive reality enhances electrophysiological correlates of implicit sensory learning

**DOI:** 10.1038/s41598-024-53937-w

**Published:** 2024-02-14

**Authors:** Pietro Sarasso, Irene Ronga, Francesca Piovesan, Paolo Barbieri, Elena Del Fante, Daniela De Luca, Ludovico Bechis, Anna Osello, Katiuscia Sacco

**Affiliations:** 1 BIP (BraIn Plasticity and Behaviour Changes) Research Group, Dipartimento di Psicologia, Università degli Studi di Torino, Via Verdi, 10, 10124 Turin, Italy; 2 VR@POLITO, Department of Structural, Geotechnical and Building Engineering (DISEG), Polytechnic University of Turin, Turin, Italy

**Keywords:** Co-presence, Shared attention, Virtual reality, Mismatch negativity, Human behaviour, Learning and memory

## Abstract

Shared attention effects on learning and memory demonstrate that experiences are amplified when we are not alone. Virtual reality poses new challenges to the study of co-presence. Above all, is coattending together with someone else’s avatar in an immersive VR setting comparable with shared experiences at a neural processing level? In the present study we investigate shared attention effects in VR for the first time. We recorded mismatch negativities (MMN) during an auditory roving paradigm, a well-known index of implicit perceptual learning. EEG responses to deviant and standard sounds were registered while subjects were alone (*Solo* condition) or together (*Other* condition) with a virtual avatar (*Virtual* scenario) or physically present confederate (*Physical* scenario). We found an overall main effect of co-presence on MMN revealed by a point-by-point 2 × 2 ANOVA, thereby replicating previous studies on physical co-presence. Additionally, we found no significant interaction between the scenario (*Physical* vs. *Virtual*) and co-presence (*Solo* vs. *Other*). Our results indicate that virtual immersive co-presence mimics physical co-presence.

## Introduction

From a philosophical point of view, embodiment, intertwining intercorporeality^1^, togetherness and spatiality have long been central issues in the phenomenology of presence^2^ and spatiality^3^ leading thinkers to ask themselves what it means to be present in space. As an example, Heidegger thought that “being present” amounts to “being-with” or “being ready-to-hand^4^. More recently, the development of digital tools and virtual reality expanded the possibility for virtual presence and re-opened this classical debate on how people experience their presence in the world (i.e., how the experience of presence is created in a virtual world^5^) and the co-presence of others^6^. Indeed, the progressive shift of human experience toward an on-line immersive virtual world opens new questions: what does it mean to be together, to which extent does it amount to being in a shared embodied space? What does “being present” mean? Is it the same to play, study or communicate with some one else who is physically or virtually present?

The physical co-presence of co-attendants has been demonstrated to be a crucial factor in perceptual processes^7,8^, so that learning and memory are enhanced^9^ and feelings and sensing are amplified when experiences are shared^10–13^. Indeed, humans might have evolved to favour information that is coattended^14,15^. Indeed, sharing mental states with peers and ingroup members favours the encoding of novel information, emotions and sensations that undergo deeper processing^16,17^. However, it is still object of debate whether the virtual co-presence (e.g. via virtual meetings or in VR) mimics physical co-presence or else if the cognitive system interprets the virtual presence of others as being alone^6^. Previous studies suggest that virtual co-presence can trigger shared attention effects. In a series of studies, Shteynberg et al.^9^ demonstrated that experience that is shared with a sham similar virtual other (the authors falsely told their participants that another participant was participating online) can intensify emotions^13^ and the recall of a list of co-attended words^18^. Coherently, more naturalistic studies have shown that online learning of factual information is enhanced for live interactive presentations than recorded video presentations^19^.

Other studies directly and systematically comparing virtual and physical co-presence^6,11^, however, found opposite results. In our previous study we showed that physical but not virtual presence of others (i.e., a video call) potentiates spatial memory and perceptual learning^6^. We compared the auditory mismatch negativities (MMN), an electrophysiological correlate of perceptual learning^20,21^, between the solo and the co-presence conditions. The MMN response to novel sensory events, is a differential wave obtained by subtracting the neural response to repeated stimuli from that of deviant (i.e., different) ones. The MMN, when elicited by sounds, results from the activations of the posterior auditory cortex and the inferior frontal gyrus^22,23^ and indexes the update of the predictive models of the sensory environment^24,25^. We found that MMN were enhanced by the physical co-presence only, while perceptual learning of virtually shared stimuli via an on-line call did not significantly differ from the solo condition^6^. In other words, we demonstrated that only the physical co-presence of others was able to increase the neural resources devoted to process novel incoming stimuli (i.e., sensory upweighting), resulting a greater update of predictive representations of the environment. Altogether, our previous study suggests shared attention effects and its underlying neural correlates are not triggered by on-line virtual co-presence.

Considering such controversial findings, it would be crucial to identify the factors responsible for the presence *vs.* absence of shared attention effects in virtual settings. Virtual co-presence is possibly situated on a continuum between solitude and physical co-presence and the position along the continuum might be related to the so called “closeness” factors. Indeed, “psychological closeness”^12^ between co-experiencers moderates the amplification of shared experiences^11^. Boothby et al.^11^, investigated psychological closeness by manipulating social (strangers vs. friends) and physical distance (being in the same physical room vs. being in different rooms connected via live video) and found that only psychologically and physically closer co-attendants showed amplified shared experiences.

The degree of embodiment and immersiveness of the shared space might be another crucial factor modulating shared perceptual experiences^26,27^. To our knowledge, however, there is no empirical study of shared attention effects and its electrophysiological correlates in virtual immersive reality. In the present study we recorded auditory MMNs during a roving paradigm to compare shared attention effects during physical co-presence and virtual immersive co-presence of an avatar in a “Oculus Rift” VR setting. We employed a 2 × 2 within-subject design. Participants performed the same EEG MMN task alone (“Solo” condition) and with a confederate (“Other” condition) both in the physical scenario and virtual room scenario. We expected: (i) to replicate previous results showing larger MMN during physical co-presence compared to the Solo condition; (ii) observe similar shared attention effects of physical and virtual immersive co-presence (i.e. null interaction effect between scenario and co-presence) if the embodiment of a VR environment can effectively reduce psychological and physical distance. More specifically, coherently with our previous study on physical and virtual non-immersive co-presence^6^, we expected to find a significant modulation in EEG signals at around 150–250 ms post-onset centred over frontal electrodes, corresponding to the MMN, when comparing waveforms registered during the *Solo* and the *Other* conditions, with a positive main effect of co-presence. Moreover, if virtual immersive reality does not induce a similar effect, as it was for virtual non-immersive co-presence, we expect to observe a significant interaction effect (co-presence × scenario).

## Results

**Figure 1:**
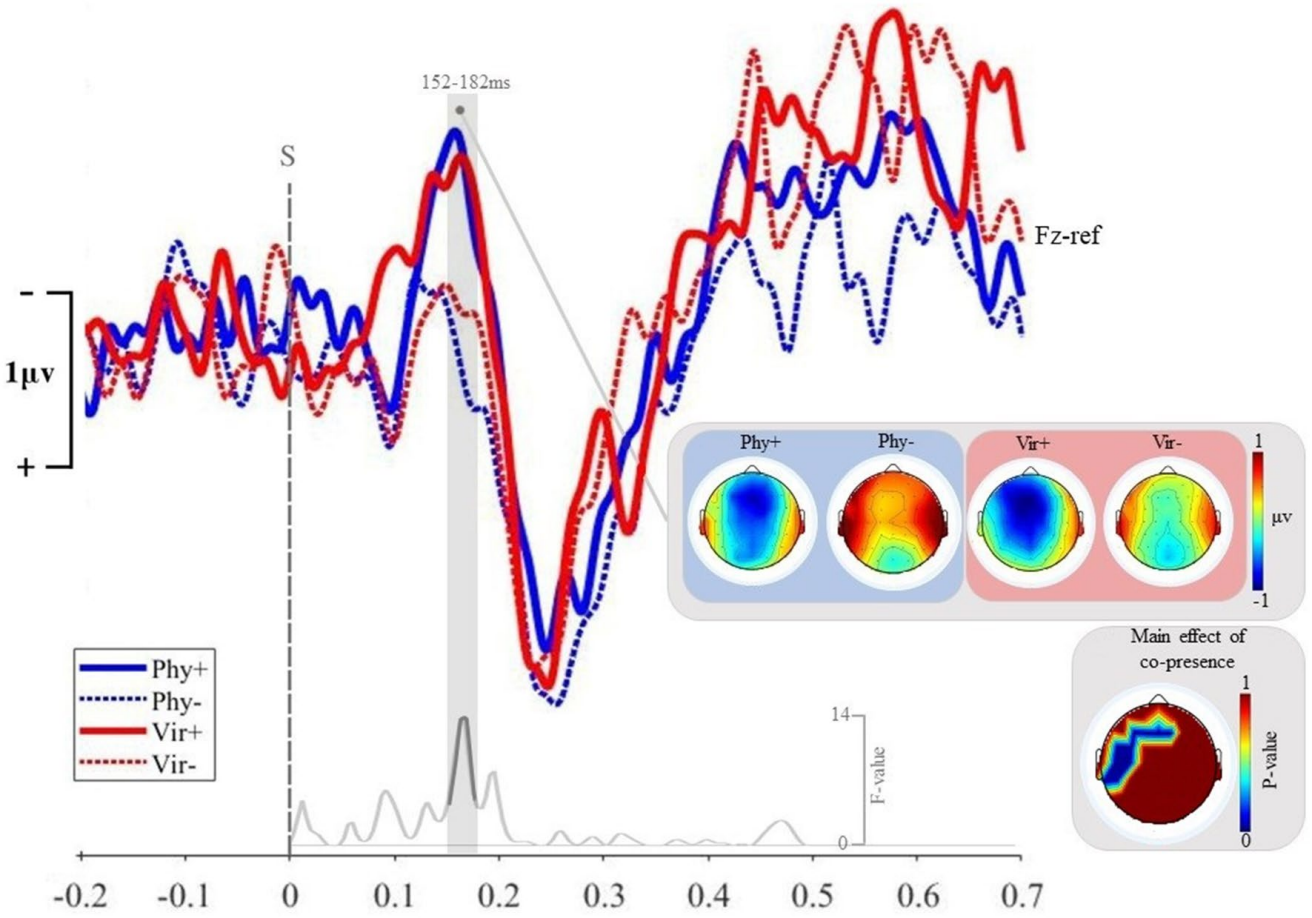
MMN waveforms. The graph represents MMN differential waveforms obtained by subtracting average responses to standard sounds from response elicited by deviant sounds at Fz. Coherently with previous studies the negative peak of MMN waveforms is frontally distributed and occurs at around 170 ms post-onset. Blue and red lines correspond to the *Physical* and *Virtual* scenario, respectively. Solid and dashed lines represent MMN corresponding to the *Other* and *Solo* conditions, respectively. The grey shaded area at 152–182 ms, corresponding to the MMN peak latency, represents the significant cluster surviving cluster correction in the point-by-point ANOVA and evidencing a significant main effect of experimental conditions *Other* vs. *Solo.* The grey line at the bottom depicts the corresponding F-values, showing a peak effect of condition corresponding to the MMN negative peak latency. Scalpmaps at the top represent the MMN amplitude distribution across the scalp for each single experimental condition. The scalpmap at the bottom shows cluster-corrected p-values corresponding to the main effect of co-presence (*Solo* vs. *Other*) resulting from the point-by-point ANOVA. The resulting significant cluster (152–182 ms) is localized over frontal electrodes overlapping the MMN scalp distribution.

Here we investigated whether and how implicit perceptual learning is modulated by contextual factors, such as the presence vs. absence of another person (or avatar) in virtual and physical reality settings. For both Other and Solo conditions, the MMN differential wave obtained by subtracting Standard from Deviant average response, showed a negative peak over frontocentral at approximately 160 ms post-onset, coherently with previous findings^6,28,29^ (Fig. 1).

Crucially, the analysis highlighted a significant effect of the factor co-presence at 152–182 ms post-onset. MMN registered while attending stimuli together with a physical or virtual co-attendant were significantly larger than those corresponding to the *Solo* condition (Fig. 1) within a time cluster corresponding to the MMN peak latency over the F1, F2, F3, Fc3 and Fz electrodes.

The main effect of scenario (*Physical* vs. *Virtual*) did not significantly modulate differential MMN waveforms. No significant cluster survived cluster-based correction for multiple comparison.

Crucially for our aims, the interaction effect was not significant at the MMN peak latency, thereby confirming that shared attention effects on the electrophysiological correlates of implicit perceptual learning of sensory regularities are comparable in the *Virtual* and *Physical* scenarios. Moreover, unexpectedly, the effect of the interaction between co-presence and scenario revealed a late right frontal cluster surviving cluster correction spanning across F4, F6, F8, Fp2, Fc4, Af4 and Af8 at 317–339 ms post-onset. As shown in Figures A and B in the supplementary material, this cluster corresponds to the P3b right-lateralized^30,31^ frontoparietal component, generally peaking slightly after the P3a component in response to pitch deviant tones^32^. Coherently with previous studies (see Polich for a review^32^) the P3b component is elicited by frequency deviant sounds only. Indeed, the P3b wave reflects a non-linear endogenous attention-dependent amplification or “ignition” of neural activity through a network involving frontoparietal areas^33^ and is generally thought to reflect post-perceptual^34^ memory updates following global violations of auditory regularities^32^. In the present study, in the Vir−  condition only, the P3b components fails to emerge after the presentation of deviant sounds.

## Discussion

The main result of the present study reveals a main effect of co-presence on fronto-parietal auditory MMN responses. This result is coherent with previous studies showing that shared attention effects are paralleled by an increase in MMN when participants are not alone. Furthermore, in the present study, we investigated for the first time shared attention mechanisms in immersive VR. Crucially, we found that shared attention effects on MMN in an immersive VR scenario were comparable with electrophysiological modulations triggered by physical proximity of a confederate, as revealed by the absence of significant interaction effects on MMN waveforms. This means that the brain interprets the co-presence of a virtual avatar in an immersive virtual environment as being together with someone else, contrarily to what we found in our previous study where the co-presence of a confederate connected via videocall did not modulate MMN responses^6^. Interestingly, at later latencies, we found a significant interaction effect corresponding to the P3b component. Deviant stimuli failed to evoke a clear P3b response in the Vir−  condition (see Fig. B in the SI). While P3a originates from stimulus-driven frontal attention mechanisms during task processing (i.e. the automatic reorienting of attention to deviant stimuli), the P3b originates from temporal-parietal norepinephrinergic activity underlying subsequent post-perceptual^34^ memory processing (see Polich^32^ for a review) requiring top-down endogenous attention to stimuli^35^. We could speculate that participants were paying less attention to the presented stream of sounds in the Vir−  condition, maybe due to the distracting novelty of the immersive VR environment, whereas co-attended deviant stimuli drew enough attentional resources to elicit a P3b component even in the virtual (distracting) environment.

In our previous study^6^ we suggested that MMN enhancement for stimuli that are co-attended together with physically co-present confederates are likely due to attentional modulations via the upweighting of post-synaptic gain in pyramidal cells encoding prediction errors that violate expectations^36^. Indeed, predictive coding^37^, which is currently the most accredited theory to account for sensory learning phenomena such as the MMN^21^, explains endogenous and exogenous attention as the modulation of precision of prediction errors and the underlying synaptic gain modulation of superficial pyramidal cells and inhibitory interneurons at different levels of the predictive sensory processing hierarchy^38,39^. Coherently with this framework of interpretation, in our previous study, we demonstrated that the information-theoretic index of prediction errors Bayesian Surprise (an index computing the information content conveyed by single stimuli as the magnitude of the update of predictive representation^40,41^) is more strongly correlated with trial-by-trial MMN responses during co-attended sound presentation. As previously discussed^6^, this means that novel (i.e. surprising) information impacts pre-existing representations more strongly when such information is shared. I.e., the cognitive system is dispositionally more likely to learn when it is sensorily coupled with others in a proximal space.

We previously investigated MMN correlates of shared attention effects during physical and virtual non-immersive co-presence registered while participant listened to an auditory roving paradigm that exactly matched the present study^6^. We found that virtual co-presence of a confederate via videocall could not modulate MMN responses as physical co-presence did. How can we explain the difference between virtual immersive and non-immersive co-presence? Coherently with previous studies^9^, we attributed this result to the modulatory effect of “closeness factors” such as physical and psychological distance Virtually present confederates on videocall might have been interpreted as too distant to increase the salience value of co-attended stimuli^6,12^. To this respect, VR introduces an unprecedented condition: the physical body of the confederates are far in space, but their avatars are next to the participant in a common embodied immersive virtual space. Our present results seem to indicate that sharing a virtual immersive space is enough to trigger shared attention effects comparable with physical co-presence effects, presumably by overcoming psychological distance.

Sharing a common (virtual or physical) embodied space might be a necessary condition for our brain to actually “feel like being together”. This highlights the importance of immersivity and embodiment in a wide range of human activities involving learning and change that are increasingly shifted on virtual platforms, such as education^26^ and psychotherapy, where the presence of others has an essential function^42^. Indeed, *feeling of presence* (i.e. feeling of “being there”^5,26^) and *embodiment* are already commonly regarded as the most important affordances provided by VR that must be considered in the design of educational immersive VR platforms^26^. Future studies should address these factors in shared VR experiences.

## Methods

### Participants

Eighteen healthy right-handed subjects participated in Experiment 1 (11 females; mean age: 25.82 years, ± 2.08 SD; mean years of education: 17.9, ± 1.54 SD). Participants were homogeneous in terms of education; most were graduate university students. All participants gave their written informed consent to participate in the study, which conformed to the standards required by the Declaration of Helsinki and was approved by the Ethics Committee of the University of Turin (Prot. n. 121724—01/03/18). Participants were not compensated for taking part in the experiment.

Sample size (N = 18) was a priori determined following Brysbaert’s guidelines^43^ which suggest that when power is unknown (there is not an established method for estimating the required sample of properly powered point-by-point cluster-based statistics; see also Sarasso et al. 2022; ^6^) one should to assume a medium effect sizes (d = 0.5), and run the required amount of participants according to the specific design, which in our case is a repeated measure test of a main effect (see Table 7 in Brysbaert^43^; required sample = 18; required power = 0.8). The resulting required sample was identical to the ones in previous studies where we employed the same EEG analyses^44^ to evaluate the effect of physical and virtual (non-immersive) co-presence on MMN^6^. 2 subjects showed poor signal-to-noise ratio (more than 20% of trials had to be rejected after visual inspection) and were excluded from group-level analysis. The final sample was thus equal to N = 16.

### Stimuli and experimental design

**Figure 2:**
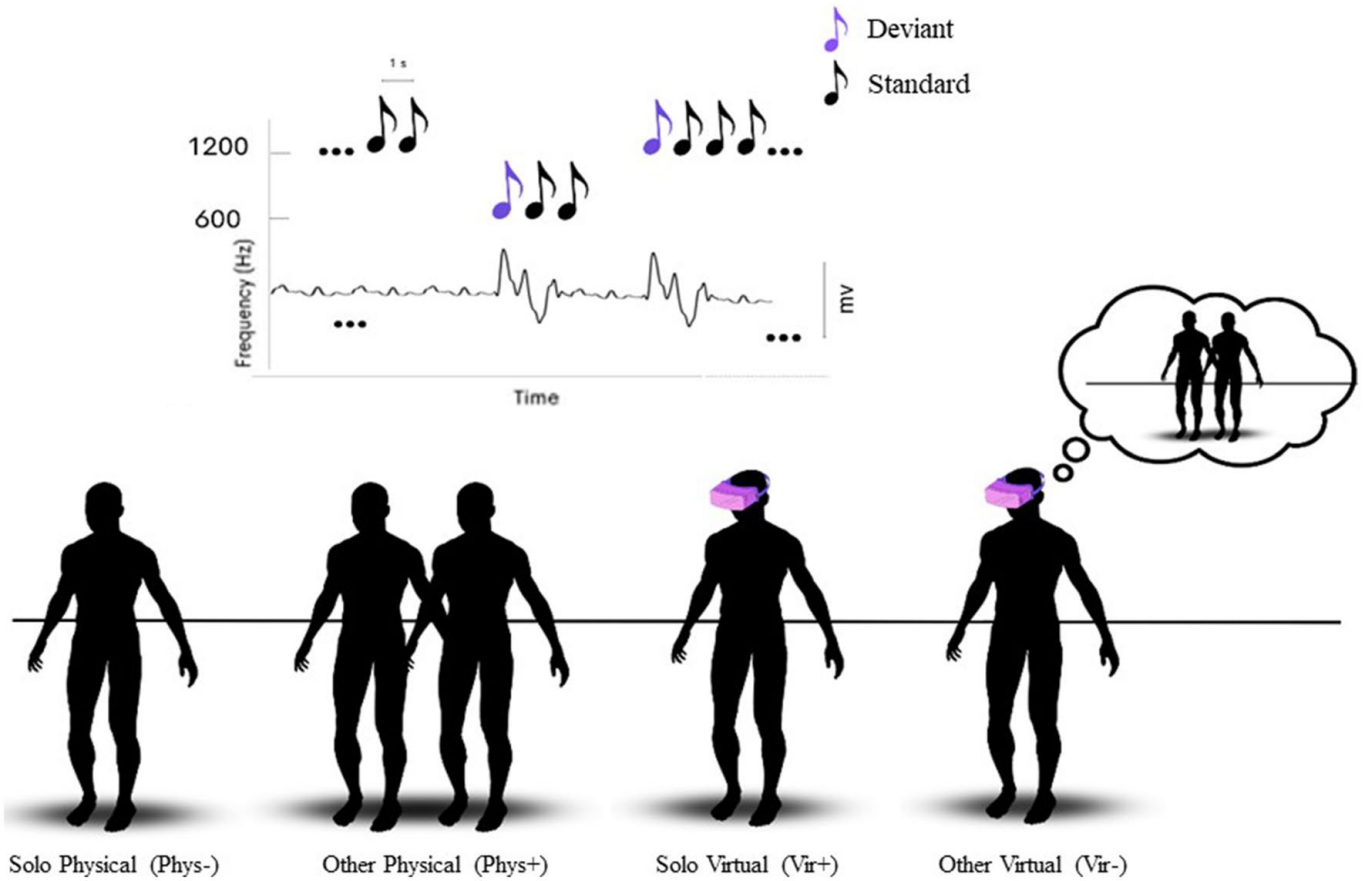
Experimental conditions and MMN task. In roving auditory paradigms high-pitch and low-pitch intervals can represent both Deviant and Standard stimuli as shown in the top panel. MMN responses are elicited by sounds deviating from a sequence of repeated sounds. Human figures at the bottom represent the 4 experimental conditions. The order of the physical and virtual sessions was randomized across participants.

The experiment was based on a within-subject design and the order of presentation of different conditions (Solo vs. Other and Physical vs. Virtual reality) was fully randomized across participants. Each participant performed the experiment in a single session lasting about 2 h. The experiment (Fig. 2) consisted of four runs, grouped into two different scenarios (i.e., *Virtual* and *Physical*). In the *Solo Physical* condition participants were alone in the experimental room during the tasks. In the *Other Physical* condition, subjects stood next to another individual. In the virtual reality setting participants wore an Oculus Rift headset and controller and navigated in a virtual environment modelled by one of the authors (DD) and loaded on Prospect-Iris VR platform^45^. Prospect by Iris VR is a commercial data modeling software for immersive VR for multiple users to collaborate. As an example, it is commonly used by the building /architecture industry to walk through 3D files (projects) with colleagues in VR. Our model was a virtual university campus classroom with a desk at the centre of the classroom designed by VR@POLITO research group. Two loudspeakers were placed (i.e. modelled) above the desk so that experimental sounds seemed to be played via loudspeakers. Sounds were actually played via headphones integrated in the Oculus Rift headset in the virtual conditions and via loudspeakers in the physical condition. In the latter loudspeakers were placed in front of the subject at 2 m. In the *Other Physical* condition, the confederate stood one meter away from the participant, so that they could easily hear the sounds played via the loudspeakers. Before starting each experimental condition, we checked that the intensity of the sounds coming from the headphones and the loudspeakers was perceived as similar by all participants. Both participants and confederates were asked to remain silent and fixate the loudspeakers during the experiments. In the virtual environment the position of the confederate avatar in the *Other Virtual* condition matched the position of the confederate in the physical setting exactly.

For each condition participants had to perform the same ‘EEG Mismatch Negativity task’ designed to investigate implicit perceptual learning. Converging evidence from electrophysiological studies suggests that the MMN elicited by the presentation of sounds that deviate from a pattern established by the preceding inputs^46^, is typically considered a neurobiological marker of implicit perceptual learning of sensory regularities^24,40^. If co-presence of another subject modulates implicit perceptual learning, we expect to observe significantly different MMNs in the two scenarios (replication of Sarasso et al. 2022; ^6^). Moreover, if the effect of virtual-immersive co-presence differs from physical co-presence we expect to observe a significant interaction between the two within factors: co-presence and virtual vs. physical setting.

EEG was registered while subjects listened to sound sequences. Deviant and Standard sounds were presented according to the classic roving paradigm^28,40^ with two sounds differing in their frequency (Hz; high-pitch and low-pitch).

### EEG MMN task

In each session, one for each scenario (*Virtual* vs. *Physical*), participants performed 2 runs of a standard roving paradigm while we registered their EEG activity. Sound sequences consisted of trains of 576 stimuli per run lasting 576 s (total duration of sound sequences: approximately 40 min). During the *Other Physical* and *Other Virtual* condition, participants and confederates were simply asked to remain silent, look straight ahead and listen to the sound sequences; during the *Solo* conditions, participants performed the same EEG MMN task alone (no one was in the room in the physical setting and no avatar was present next to the participant’s avatar in the virtual classroom). The order of presentation of the two settings was randomized across participants, as to exclude any specific sequence effect. Differently from traditional oddball paradigms^47^, where the repeated presentation of standard sounds is occasionally interrupted by the occurrence of physically different deviant sounds, in roving paradigms^48,49^ different stimuli (high-pitch and low-pitch intervals in our case) can represent both Deviant and Standard stimuli (Fig. 2).

High-pitch and low-pitch intervals were presented in consecutive trains of alternating pitch with a constant inter-stimulus interval of 1 s. Any time a change in the stimulation stream occurs the first stimulus of the new train constitutes a Deviant event, since it differs from the preceding train of stimuli, which are therefore considered Standard^28^. The length of the trains of high-pitch (600 Hz) and low-pitch (150 Hz) sounds was chosen according to a pseudo-random order, so that both the number of presentations and the average value of the Bayesian surprise (see Sarasso et al. 2022; ^6^) were equal across pitch types (i.e. high or low; Fig. 2). Moreover, the ratio between Standard (80%), and Deviant (20%) trials was kept constant across runs. Differently from traditional oddball paradigms, in roving protocols each stimulus type has exactly the same probability of occurrence, thus allowing to dissociate genuine effects of Bayesian perceptual learning from rarity-driven modulations^24^.

### EEG recording and preprocessing

EEG data were collected during the 4 runs of the EEG MMN task with 64 Ag–AgCl electrodes placed on the scalp according to the extended International 10–20 system and referenced to Fcz. Electrode impedances were kept below 5 kΩ. The electro-oculogram (EOG) was recorded from two surface electrodes, one placed over the left lower eyelid and the other placed lateral to the outer canthus of the left eye. EEG activity was recorded with Brain Amp DC system and continuously digitized at a sampling rate of 1024 Hz. Data collected during the EEG MMN Task were off-line re-referenced to Oz and pre-processed with Letswave6 (an open-source EEG/EMG signal processing toolbox, https://www.letswave.org/). Data were segmented into epochs of 1 s, including 200 ms pre-stimulus and 800 ms post-stimulus intervals. Epochs were band-pass filtered (0.5–40 Hz) using a fast Fourier transform filter (in accordance with previous literature exploring MMN^28,40^). Filtered epoched data were baseline corrected using the interval from − 0.15 to 0 s as reference. Ocular artefacts were eliminated using Independent Component Analysis (ICA^50^). Remaining artifacts and noisy trials were eliminated manually after visual inspection. Recordings (participants) that included at least 1 run with more than 20% rejected epochs were excluded from subsequent analyses.

### Data analysis

For each scenario (Physical vs. Virtual), ERPs belonging to the same condition (i.e., Solo or Other) and to the same type (i.e., standard vs. deviant) were then averaged, to obtain four average waveforms per scenario for each single subject (i.e., Solo Standard, Solo Deviant, Other Standard, Other Deviant). MMN differential waveforms were then computed by subtracting deviant responses from standard responses (in this analysis we included only the last standard trial for each stimuli train occurring before deviant trials^28^). We so obtained 4 MMN waveforms for each subject corresponding to the *Solo Physical*, *Other Physical*, *Solo Virtual* and *Other Virtual* conditions which constituted the input of group-level analyses.

In the present study we employed point-by-point cluster-based corrected statistical tests^51,52^. Point-by-point, cluster-based permutation tests of EEG/ERP data are widely used methodologies in the fields of psychophysiology and neuroscience^6,28,44,53–63^ directed to highlight possible differences between conditions across the whole epoch time-course, without any a-priori assumption, and are implemented by most analysis software such as Fieldtrip^64^ and Letswave^65^ (see the referenced web pages for a complete tutorial). EEG signals are characterized by a spatiotemporal structure in which virtually infinite statistical tests (like standard *t*-tests) could be conducted, one for each data sample in time; however, standard approaches (i.e., extracting peak values and running *t*-tests or ANOVAs) have been long criticised since they may lead to errors related to multiple statistical comparison^66^. Classical correction methods for multiple comparisons may reduce the measured effect size and the probability of observing the true effect present in the data^67^. Additionally, extracting peak values to evaluate amplitude difference at single latencies involves a huge loss of information that are discarded and do not contribute to the resulting statistical power, thereby causing a problem of low replicability: if a latency window and spatial region are chosen a priori, and by chance they do not coincide with the effect of interest, an experimenter will be unable to detect a true effect^68^.

To tackle these issues and maximize the power of statistical analyses, a cluster-based correction analysis has been proposed^51^. This approach assumes that neural effects span across clusters in the different dimensions of interest (space, time, and/or frequency) and further makes use of the EEG property that neural responses are clustered. Indeed, amplitudes or frequency power on adjacent scalp locations and latencies are often correlated, because a real electrophysiological effect most likely affects multiple adjacent electrodes similarly and persists across several tens of milliseconds. Point-by-point cluster-based analyses test the null hypothesis that observations corresponding different conditions are drawn from the same distribution and are therefore exchangeable. When observing similar effects under random assignment of condition labels is unlikely (less than 10% of the permutations in our case), cluster-based correction rejects this hypothesis and the observed effect is considered significant^51,69^.

In simple terms, cluster-based point-by-point analyses (a.k.a sample-by-sample) run a very high number of *t*-tests or Anovas (like in our case), one for each sample in time (so if you record with a sample rate of 1024 Hz, you will be able to compute 1024 tests per second) and then correct results based on temporal and spatial contiguity. Clusters in the observed data are then regarded as significant if their cluster p-value exceeds the threshold of a given percentile of the permutation distribution (usually corresponding to a critical alpha-level of 0.05^69^). According to this approach, one statistical comparison for each time point composing a waveform for each electrode separately was performed in the present paper. In order to correct for multiple comparisons, a cluster-based permutation test (1000 random permutations; alpha level = 0.05; percentile of mean cluster sum = 90; minimum number of adjacent channels = 5) was employed to each point-by-point analysis^3^. Significant clusters were based on both temporal contiguity and spatial adjacency of a minimum of five electrodes. The so obtained clusters of significance represent the result of the point-by-point analyses, corrected by permutation testing.

We performed a point-by-point ANOVA with two within-subject factors corresponding to co-presence (*Solo* vs. *Virtual*) and scenario (*Physical* vs. *Virtual*). The analysis computed an F and p value corresponding to main and interaction effects for each single timepoint of the waveform (at each single latency) for each electrode separately, as well as clusters of significance surviving cluster correction.

### Supplementary Information

Supplementary Figures.

## Data Availability

Raw EEG recordings will be publicly available at Mendeley repository.
