# Supplementary material for: Shared attention in virtual immersive reality enhances electrophysiological correlates of implicit sensory learning
Source: Sci Rep. 2024 Feb 14;14:3767. doi: 10.1038/s41598-024-53937-w (PMC10866920; doi:10.1038/s41598-024-53937-w)
Supplement: Supplementary file 1 — Supplementary Figures. [file 41598_2024_53937_MOESM1_ESM.docx]

**Supplementary Information**


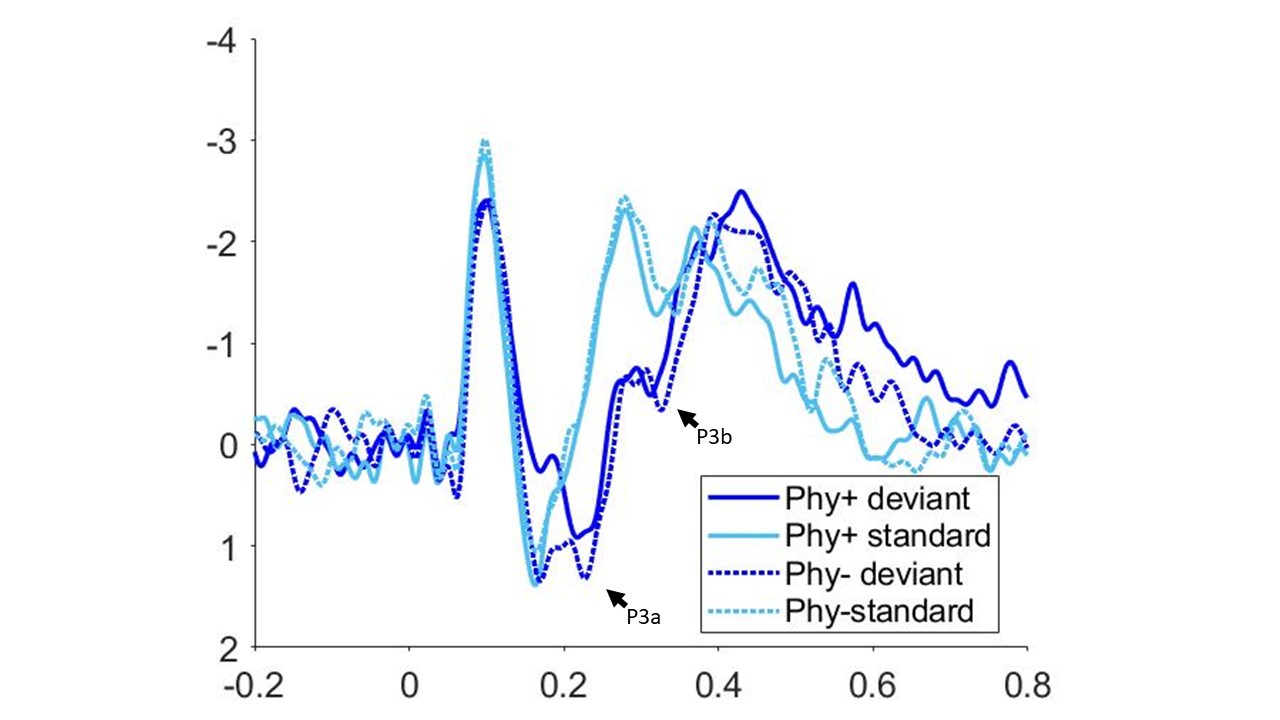


**Figure A.** Average ERPs elicited by frequency (Hz) standard and deviant sounds in the Physical scenario.


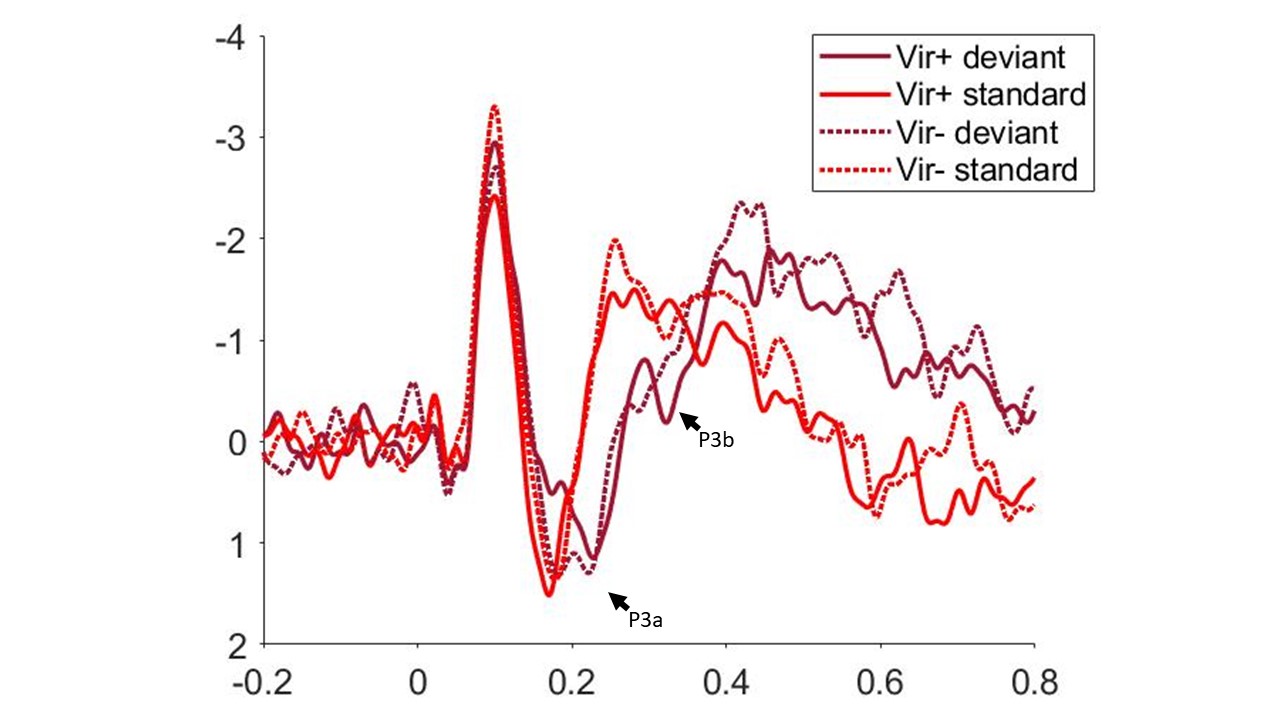


**Figure B.** Average ERPs elicited by frequency (Hz) standard and deviant sounds in the Virtual scenario.
